# RURAL DISPARITIES IN THE USE OF FAMILY AND FORMAL CAREGIVING FOR OLDER ADULTS WITH DISABILITIES

**DOI:** 10.1093/geroni/igad104.1707

**Published:** 2023-12-21

**Authors:** Katherine Miller, Katherine Ornstein, Norma Coe

**Affiliations:** Johns Hopkins Bloomberg School of Public Health, Baltimore, Maryland, United States; Johns Hopkins University, Baltimore, Maryland, United States; University of Pennslyvania, Philadelphia, Pennsylvania, United States

## Abstract

As federal and state policies rebalance long-term care from institutional settings to home- and community-based settings, reliance on formal (paid) and family (unpaid) caregivers for support at home nationally has increased in recent years. Yet, it’s unknown if use of formal and family care varies by rurality as a disproportional share of the aging population reside in rural areas. Using the Health and Retirement Study, we describe patterns in receipt of combinations of formal and family home care by rurality among community-dwelling adults aged 65+ with functional limitations from 2004-2016. Compared to older adults in urban areas, older adults in rural areas are more likely to receive any family care than those in urban areas and less likely to receive any formal care (2.4 and -4.4 percentage points respectively, p<0.05). From 2004 to 2016, a growing proportion of older adults in rural areas receive care from family caregivers exclusively while a decreasing proportion receive care from formal caregivers exclusively. When examining older adults in urban areas, we find the opposite trend — a growing proportion of urban adults rely exclusively on formal care and a decreasing proportion rely exclusively on family care. We find that national estimates of sources of caregiving and their trends mask significant heterogeneity in uptake by rurality. Understanding how older adults in rural areas are, or are not, receiving home-based care compared to their urban peers and how these patterns are changing over time is the first step to informing supports for family and formal caregivers.

